# The Role of Glia Telomere Dysfunction in the Pathogenesis of Central Nervous System Diseases

**DOI:** 10.1007/s12035-024-03947-6

**Published:** 2024-01-19

**Authors:** Manthia A. Papageorgakopoulou, Angelina Bania, Ioanna-Aglaia Lagogianni, Kyriakos Birmpas, Martha Assimakopoulou

**Affiliations:** 1 School of Medicine, University of Patras, 26504 Patras, Greece; 2 Department of Anatomy, Histology and Embryology, School of Medicine, University of Patras, Preclinical Medicine Department Building, 1 Asklipiou, 26504 Patras, Greece

**Keywords:** Glia, Telomeres, Telomere shortening, Neurodegenerative diseases, Psychiatric diseases, Gliomas, Traumatic brain injury

## Abstract

Maintaining the telomere length is decisive for the viability and homeostasis process of all the cells of an organism, including human glial cells. Telomere shortening of microglial cells has been widely associated with the onset and progression of neurodegenerative diseases such as Parkinson’s and Alzheimer’s disease. Additionally, traumatic brain injury appears to have a positive correlation with the telomere-shortening process of microglia, and telomere length can be used as a non-invasive biomarker for the clinical management of these patients. Moreover, telomere involvement through telomerase reactivation and homologous recombination also known as the alternative lengthening of telomeres (ALT) has been described in gliomagenesis pathways, and particular focus has been given in the translational significance of these mechanisms in gliomas diagnosis and prognostic classification. Finally, glia telomere shortening is implicated in some psychiatric diseases. Given that telomere dysfunction of glial cells is involved in the central nervous system (CNS) disease pathogenesis, it represents a promising drug target that could lead to the incorporation of new tools in the medicinal arsenal for the management of so far incurable conditions.

## Introduction

Telomeres constitute nucleoprotein structures composed of TTAGGG repeats forming G-overhangs and G-quadruplexes protecting chromosome ends during DNA replication which would otherwise undergo gradual shortening with each division. In that regard, telomeres, often termed the “molecular clock” of cells, shield the genome from the loss of invaluable genetic information through each replication cycle. Telomere integrity is protected by a complex of six proteins (TRF1, TRF2, RAP1, TPP1, TIN2, and POT1) called shelterin [1–3]. Telomere length shortening is a physiological process that predominantly occurs in mitotically active somatic cells due to the inability of DNA telomerase (a telomere maintenance enzyme which consists of TERC, an RNA template for DNA synthesis, and TERT, a rate-limiting subunit with reverse transcriptase, and RNA binding catalytic action) to replicate telomere ends. Eventually, they reach a point of no return, where the shelterin cap dissociates, leaving the 3′-overhang exposed to be recognized as DNA breaks, leading to cell cycle arrest or fusion with another chromosome [4]. Hence, perpetual replication is deterred in somatic cells, effectively preventing aberrant cell division and tumorigenesis, inducing replication arrest after a fixed cycle of cell divisions, known as the “Hayflick limit.” Thus, telomeres play a crucial role in physiologic cellular functions to preserve homeostasis through replication aging [1–4].

Telomere attrition is a central aspect of the telomere-cellular senescence-aging axis, resulting from semiconservative DNA replication. The consequence is genomic instability and subsequent activation of the DNA damage response pathway (DDR pathway) which drives the cell toward irreversible cell cycle arrest and the allostasis state of “senescence” [5]. Cellular senescence and telomere shortening consist typical hallmarks of cell aging, and consequently organ aging, compromising its regeneration capacity [6–8]. Stem cells like neural stem cells and neural progenitor cells maintain telomere length through a high level of telomerase activity and preserve their telomere length and proliferative capacity bypassing the Hayflick limit and achieving cellular immortality [9, 10]. Neurons are considered post-mitotic cells, incapable of dividing (although this statement’s legitimacy remains a controversial issue among scientists), whereas glial cells preserve their mitotic capability [11, 12]. Studies on normal cerebral tissues have shown that neurons demonstrate the longest telomeres and no age-related attrition contrary to telomeres of glial cells in the white matter which show age-related attrition [13]. Among CNS glial cells (astrocytes, microglia, oligodendrocytes), microglial cells are the most mitotically potent cells and thus prone to telomere shortening [14, 15].

Deciphering telomere dynamics is pivotal in unraveling the complex interplay between telomere shortening and various pathologies encountered in the CNS. The regulation of telomeres and telomerase holds significance in cancer neurobiology and age-related processes, shaping the physiological landscape of cell replication and longevity. The aim of this article is to overview the recent reports that refer to the role and possible implication of glial cell telomeres in neurodegenerative, and psychiatric diseases, gliomas, and traumatic brain injury as well as to present relevant telomere-targeting therapeutic strategies for these CNS human diseases.

## The Role of Glia Telomere Dysfunction in CNS Neurodegeneration

Microglial cells play a major role in neuroprotection and immunological surveillance of the brain and are activated in various CNS pathologies, including injury, trauma, or stroke to maintain neuronal survival by reinforcing the secretion of trophic factors and cytokines [16]. Accumulating evidence reveals that during brain aging, microglia establish gradually an inflammatory and cytotoxic environment for neurons [17] and are subject to morphological and functional transformation [18]. Moreover, these changes may be predictive of the development of neurodegenerative conditions [18]. Since telomere shortening is one of the major features of aging, this could be considered inductive to the establishment of a dystrophic microglia phenotype [19]. The exact mechanism by which telomere shortening causes microglia senescence is not well-established. Herbig et al., through their experiments in human fibroblasts, demonstrated that the overexpression of the TRF-2 mutant form led to telomere uncapping in the 3′ guanine-rich sequence [20]. This exposed region may initiate DNA damage signals, resulting in the activation of phosphatidylinositol 3-kinase (PI3K)-like protein kinases (PI3KKs), mainly ATM and, to a lesser extent, ATR. These pathways activate the p53 protein and upregulate p21CIP1, a Cdk inhibitor, causing retinoblastoma (RB) hyperphosphorylation and, consequently, cell cycle arrest in the G1 phase. When ATM-P53 is deactivated, the cell cycle initiates again, highlighting its crucial role in cellular senescence including the brain as reviewed by Liu and Saez-Atienzar and Masliah [21, 22].

One of the primary consequences of DNA damage and subsequent cell cycle exit in microglia is the transition to a senescence-associated secretory phenotype (SASP) [23–25]. In this state, microglia produce pro-inflammatory cytokines (TNFα, IL-1β, IL-6, and IL-8), influenced by the expression of NF-κΒ, ROS, proteases, and other molecules that could damage the surrounding tissue, leading to apoptosis of neurons and other glial cells. Accumulation of Aβ deposition and neurofibrillary tangles (NFTs) may cause genetic, metabolic, and DNA damage, triggering SASP not only in microglia but also in other brain cell types [17, 18, 23–25]. However, it remains controversial whether proteinopathy or cellular senescence occurs first. Indeed, Khan et al. showed that telomere dysfunction in genetically altered mice with TERC gene knock-out (KO) mutations did not correlate with microglia aging or morphological changes of microglial cells although a reduction in microglial cell number was observed [26].

Another effect of aging in microglia is the amplification of its immunogenicity, a condition known as “priming” [27]. Microglial priming induces a neurodegenerative phenotype which demonstrates great relevance to Alzheimer’s disease. Specifically, primed microglia can induce excessive neuroinflammation and accumulation of neurofibrillary tangles due to phosphorylation of tau protein and production of amyloid β (Aβ) disrupting thus neuronal integrity as well as reduction of the release of neurotrophic factors resulting further in loss of normal neurons [28].

Raj et al. examined in a mouse model of telomere shortening whether telomere shortening could initiate an inflammatory response activating microglia and revert it to priming condition [29]. It is well-known that telomere shortening leads to the re-localization of RAP1—a protein structural component of telomeres—from telomeres to extratelomeric genomic sites which may affect gene transcription [30]. Indeed, mice expressing extratelomeric RAP1 show increased production of pro-inflammatory cytokines and chemokines through the NF-kb pathway in brain tissues [31]. However, Raj et al. concluded that telomere shortening does not impair the phenotypic and functional properties of microglia and does not result in microglia priming [29]. Thus, the exact role of microglia telomere dysfunction in CNS neurodegeneration remains elusive, and further research is necessary to clarify this association.

## The Role of Microglia Telomere Shortening in Alzheimer’s Disease

It was hypothesized that telomere shortening is paramount in the onset and progression of neurodegenerative diseases such as Alzheimer’s and Parkinson’s disease through mechanisms that impede the function of glia, especially microglial cells [32]. Alzheimer’s dementia (AD) is a neurodegenerative disorder that has been inextricably linked to aging [21, 22]. It is characterized by cognitive impairment, neural cell death, and loss of neuronal synapses due to Aβ amyloid accumulation and neurofibrillin tangles, aggregates of hyperphosphorylated tau proteins [33].

Microglia senescence is considered a major risk factor for AD maintenance and progression, as glial cells lose their neuroprotective capacity during this state [15, 32]. Rolyan et al. using transgenic mouse models demonstrated that, even though telomere shortening is correlated with aged microglia and was expected to precipitate the progression of the disease, it impeded the neurodegenerative phenotype and Αβ plaque accumulation [34]. However, Hu et al. provided evidence of AD pathology associated with a group of senescent microglia cells (the so-called diseased-associated microglia) and that microglial proliferation (linked to telomere shortening) constitutes a risk factor for neurodegeneration. Early-stage inhibition of microglial proliferation in AD-like pathological mice impaired the onset of microglia senescence and diminished amyloid-induced pathology [35].

Given that deficiency of folate, the co-enzyme involved in homocysteine metabolism and nucleotide synthesis is associated with neurodegenerative disorders, including AD, folic acid (the therapeutic form of folate) could be a therapeutic approach for these disorders [36]. Interestingly, recent preliminary in vitro data demonstrate that folic acid decreases astrocyte apoptosis by preventing DNA oxidative stress-induced damage and telomere attrition [37]. Although further studies are needed to confirm these findings in vivo, this study introduced a link between folate deficiency and astrocyte telomere dysfunction in AD [37].

## The Role of Microglia Telomere Shortening in Parkinson’s Disease

Parkinson’s disease (PD) is a multifactorial neurodegenerative disorder with a prevalence of 1% in individuals older than 60. The predominant pathophysiological feature of PD is the neuronal loss in the dopaminergic nucleus pars compacta at the substantia nigra, bearing the typical histological finding of Lewy bodies [38]. Five major genes have been associated with familial PD, the most important are the synuclein gene (SNCA), the parkin gene (PARK 2), and the ubiquitin C terminal hydrolase L1 gene (PARK 5). Crucial in the disease pathogenesis is the aggregation of misfolded synuclein proteins within Lewy bodies and Lewy neurites [38].

The association of PD with aging among other established risk factors unfolds a promising landscape to investigate the role of cellular aging in its pathogenesis. A study conducted by Scheffold et al. in a PD mouse model with telomere shortening phenotype (TERC knockout) indicated that earlier onset of synuclein aggregation had taken place with documented defective microglial immune response in the aged brain [39]. Thus, the study introduces the contribution of microglia telomere attrition in PD pathogenesis and adds to the hypothesis that telomere erosion with aging may be a risk factor in neurodegenerative disorders [40].

## The Role of Glia Telomere Dysfunction in Glioma Pathogenesis

### Genes Involved in Telomere Regulation Are Implicated in Gliomagenesis

According to a case–control study by Wang et al., telomere length in peripheral blood leukocytes correlates with glioma risk [41], which has also been proven in the Mendelian Association and Genome-Wide Association Studies [42–44]. More specifically, these studies attribute an increased risk for the development of glioma to genetically increased leukocyte telomere length and identify also numerous polymorphisms in telomere-regulating genes, including, among others, members of the shelterin complex, such as POT1, and the TERT and TERC subunits of telomerase itself. Another notable identified glioma risk locus regarding leukocyte telomere length is PARP1, known not only for its role in DNA repair but also for its ability to regulate the binding of TRF2 (Telomeric repeat-binding factor 2), a shelterin subunit, to telomeric DNA [45]. Additionally, SNPs in the RTEL1 locus—a helicase heavily implied in telomere maintenance—have been associated with high-grade glioma risk [46].

## The Role of TERT Promoter and ALT Pathway in Gliomagenesis

**Fig. 1:**
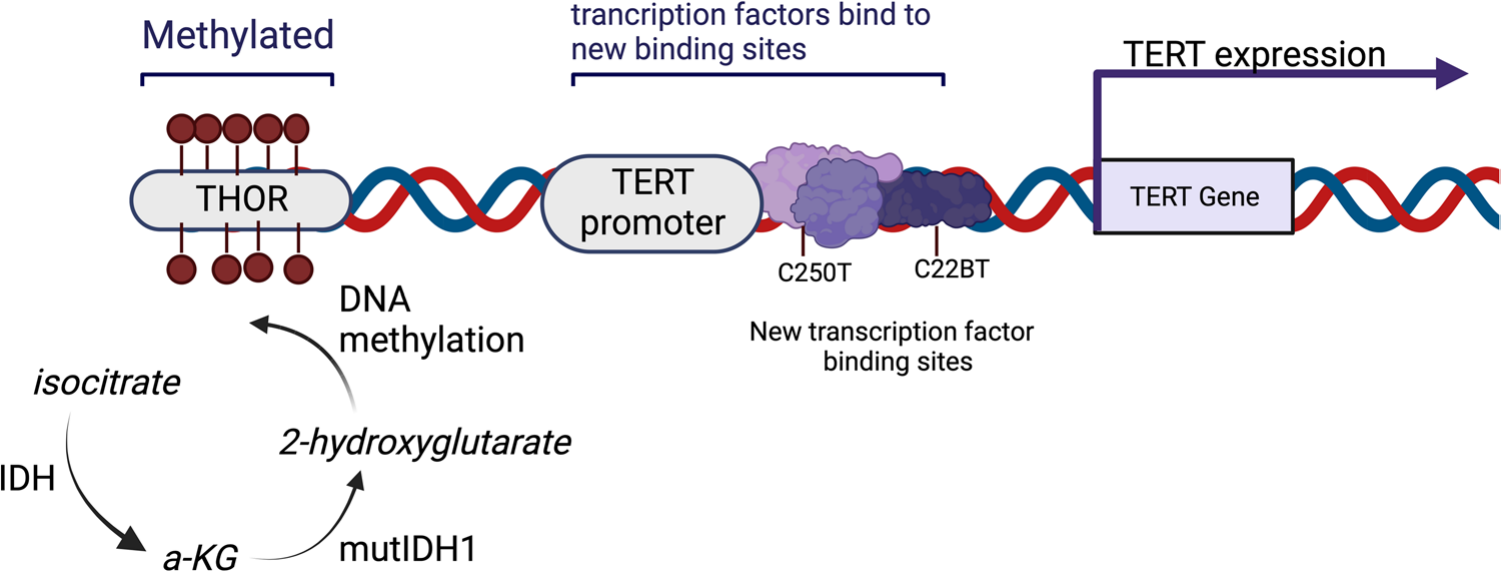
Telomerase re-activation as a telomere elongation pathway in cancer. C250T and C228T are the two most common TERT promotor mutations that lead to the creation of new transcription factor binding sites, thus promoting telomerase expression. THOR (TERT hypermethylated oncologic region) is located upstream of the TERT promoter. Its hypermethylation can be enhanced by 2-hydroxyglutarate produced from α-ketoglutarate (α-KG) by mutant IDH1 and induces TERT transcription (created with Biorender.com)

Telomere alterations related to gliomagenesis take place via two main pathways: (i) telomerase reactivation and (ii) alternative lengthening of telomeres (ALT). Telomerase reactivation occurs mainly via changes on the TERT gene promoter. Mutations on the TERT promoter itself, among which C228T and C250T are the most common in CNS malignancies, have been shown to create new binding sites for transcription factors, activating, thus, the telomerase synthesis pathway [47]. Additionally, changes outside the TERT gene and its promoter can increase TERT expression, as is the case of THOR (TERT hypermethylated oncological region) hypermethylation [48] (Fig. 1).

Isocitrate dehydrogenase (IDH) exists in three isoforms (IDH1, IDH2, IDH3) and normally converts isocitrate to α-ketoglutarate (α-KG). When IDH is altered (usually via the R132H gene mutation), it generates 2-hydroxyglutarate (2-HG) from α-KG, which participates in abnormal DNA methylation and ROS-mediated damage. These alterations have been related with tumorigenesis in gliomas [49–51]. IDH1 mutations occur in lower-grade astrocytomas and secondary glioblastomas affecting young patients with a generally good prognosis [52]. It has been found that mutant IDH1 astrocytes exhibit an infinite replication potential due to telomere stabilization and high levels of telomerase and c-Myc/Max transcription factor binding on the TERT promoter [51, 53]. Interestingly, epidermal growth factor receptor (EGFR) inhibition in human glioblastoma cells results in a reduction of telomerase activity and telomere shortening and is associated with slower tumor progression in mice in a dose-dependent manner [54].

**Fig. 2:**
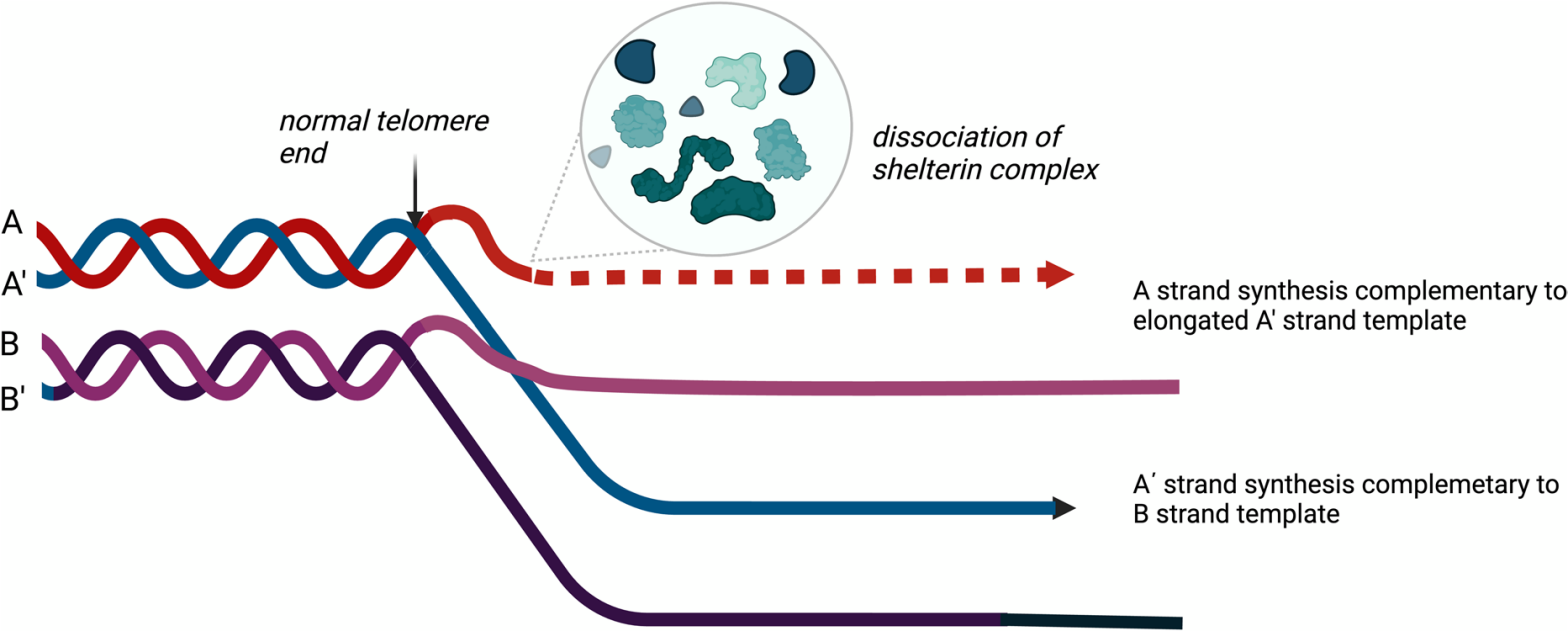
The alternative lengthening of telomeres pathway in cancer. Loss of shelterin proteins exposes telomeres and allows them to interact with other DNA strands. Homologous recombination at these sites can then occur between sister chromatids which leads to DNA synthesis at telomere ends and thus telomere elongation (created with Biorender.com)

The ALT pathway accommodates a telomerase-independent, telomere expansion via homologous recombination (Fig. 2). The ALT phenotype includes ALT-associated PML (promyelocytic leukemia) bodies (APBs) on recombination sites, extrachromosomal telomeric ssDNA (C-circles) and telomeric sister chromatid exchange (T-SCEs) [55]. One of the main mutations found in the ALT phenotype is the inactivation of ATRX, which participates in the ATRX/DAXX (alpha-thalassemia/mental retardation syndrome X-linked/death domain-associated protein) chromatin remodeling complex. IDH1 mutations can directly suppress ATRX expression resulting in ALT [56]. Experiments in SV40-transformed fibroblasts indicate that ATRX knockdown increases the percentage of culture immortalization, whereas combined ATRX and DAXX downregulation reduces the time required for immortalization [57]. Thus, ATRX may be an ALT repressor; furthermore, loss of ATRX function cooperates with one or more as-yet-unidentified genetic or epigenetic alterations to activate ALT [57]. ATRX loss has been shown to be associated with ALT in adult and pediatric GBMs [56, 58]. Additionally, IDH1 mutation is necessary for the induction of the ALT phenotype in p53/pRb-deficient ATRX-knockout astrocytes, which acquire the feature of growth on soft agar (a hallmark of glioma) [59]. Moreover, mutant IDH1 is associated with RAP1 (Ras-proximate-1) gene and XRCC1 (X-ray repair cross-complementing 1) gene (responsible for DNA break repair and non-homologous end joining) downregulation which have been proven to play a role in driving the ALT phenotype during gliomagenesis [59].

Additionally, general control non-depressible 5 (GCN5) and P300/CBP-associated factor (PCAF) are two largely homologous proteins with opposite effects on the alternative lengthening of telomeres process in human glioma cells [60]. GCN5 binds to ubiquitin-specific peptidase 22 (USP22) which deubiquitinates and thus salvages the telomeric repeat-binding factor 1 (TRF1) shelterin protein. Therefore, telomeres are protected from undergoing structural changes and homologous recombination. Conversely, PCAF increases telomeric instability and may regulate ABP formation, inducing the ALT phenotype [60].

It is worth noting that the activation of the telomere maintenance mechanism (TMM) is dynamic and reflects the plasticity and oncogenicity of tumor cells under internal and external pressures to survive. Thus, TMM in gliomas cannot be defined solely by the combination of telomerase activity and ALT. In fact, during the progression of the disease, gliomas have the capacity to switch from one telomere maintenance mechanism to another [61]. In a comprehensive retrospective cohort study by Kim et al., the TMM landscape unfolded across distinct groups—telomerase-positive, ALT-positive, negative for both telomerase and ALT, and a subset exhibiting both activities contributing to profound intratumoral heterogeneity. Contrary to conventional assumptions, neither TERT promoter mutations nor ATRX loss reliably predicted TMM categories. These findings underscore the intricate and evolving nature of TMM in gliomas suggesting a complex interplay of factors influencing TMM [61]. Importantly, recent findings in pediatric high-grade gliomas (pHGGs) like diffuse intrinsic pontine glioma (DIPG), and telomere maintenance mechanisms exhibit a noteworthy heterogeneity. The coexistence of telomerase and ALT pathways within the same tumor underscores the intricate nature of intratumoral heterogeneity in telomere length and maintenance mechanisms [62].

## Diagnostic and Predictive Significance of Telomere Biology in Gliomas

It is of vital importance to differentiate between histologic and genetic subtypes of brain tumors that carry different prognoses and treatment options. In terms of diagnosis, the mutational burden of genes relevant to telomere regulation can be highlighted in tumor histology. Such genetic signatures have been proven useful in the histologic and prognostic classification of gliomas. More specifically, I-CF (IDH1/CIC/FUBP1) tumors are of oligodendroglial origin and carry IDH and at least one of CIC, FUBP1 (far upstream element binding protein 1) or 1p/19q mutations, whereas ATRX and IDH alterations define I-A (IDH1/ATRX) tumors, characterized by astrocytic histology, ALT mutations, and younger age at diagnosis. Furthermore, a heterogeneous category of IDH and ATRX wild-type tumors like glioblastoma multiforme (GBM) was defined as I-X [63]. More recently, analysis of ependymomas suggested that only the loss of ATRX immunoreactivity may be used as evidence against the diagnosis of ependymoma, as these tumors do not exhibit the ALT phenotype [64].

Telomeric G quadruplexes are nucleic acid structures formed between guanine bases and may represent a reliable biomarker in tumors since their formation is more pronounced in the ALT pathway [65]. This enables distinguishing low-grade gliomas in terms of their telomere maintenance mechanism (TMM) in a quick and reliable manner, using Naptho-Template assembled synthetic G-quartet (N-TASQ) probes through immunohistochemistry. ALT-dependent glioma diagnosis could also be achieved by c-circle assay, with the additional advantage of low DNA requirements [66]. 1H-magnetic resonance spectroscopy represents another promising non-invasive strategy, by detecting biomarkers specific to the tumor’s genetic subclassification or benign findings such as gliosis. Hyperpolarized [1-13C]-alanine is metabolized in vivo in either lactate, when the TERT pathway is activated, or pyruvate which is indicative of the ALT pathway [67].

Contrary to previous reports [68, 69], recent data show that activation of the ALT pathway or failure to identify a specific telomere maintenance mechanism is associated with poorer prognosis for glioblastoma patients [70]. However, ATRX loss associated with the ALT phenotype may be another promising marker with prognostic significance, as it is the hallmark of a favorable type of IDH-mutant astrocytomas [71, 72]. Moreover, recent findings introduce the potential existence of different types of ALT based on the level of activity of the pathway as identified by c-circle since the younger patients (25–30 years) with IDH-mutant ATRX-lost anaplastic astrocytoma display higher intensity of ALT compared with 66–70 years-old patients [73]. On the other hand, glioblastomas having progressed from anaplastic astrocytomas do not demonstrate this correlation [73]. Interestingly, further data show the development of ALT without ATRX loss in a subgroup of pediatric high-grade gliomas introducing germline variants in mismatch repair (MMR) genes as the possible cause of increased occurrence of ALT in these patients [74]. In this line, a study in pilocytic astrocytomas demonstrates that ALT-positive/ATRX-negative anaplastic pilocytic astrocytomas are a distinct category of gliomas which is associated with worse overall and recurrence/progression-free survival [75].

TERT promoter mutational status has a prognostic impact in gliomas which is different among the various subtypes of gliomas [47, 72]. Specifically, TERT promoter mutation has been significantly associated with worse survival in IDH-wildtype astrocytomas. In contrast, TERT mutation has been significantly associated with better survival in IDH-mutant astrocytomas and 1p/19q-codeleted oligodendrogliomas [72, 76]. Nevertheless, novel data define molecular subgroups of IDH-wildtype glioblastomas, including a telomerase-positive subgroup driven by TERT structural rearrangements and an ALT-positive subgroup with mutations in ATRX or SMARCAL1 which demonstrate poorer survival compared with IDH-mutant/TERT-wildtype GBMs [77]. In addition, certain TERT polymorphisms are associated with an increased risk of developing GBM, and an association between somatic TERT promoter mutations also results in a reduced OS of patients with primary glioblastoma [78]. Long telomere length and the TERT mutations C228T and C250T are not only indicators of poor survival but also of radioresistance in gliomas [79]. In a previous cohort however, the unfavorable prognosis of glioblastoma patients with TERT activation had been attributed to the older age of the patients and not to the subsequent telomerase activation per se [80]. Importantly, recent evidence confirms the relationship between telomerase activation and the older age of patients with gliomas [61]. Although TERT promoter mutations are extremely rare in pediatric gliomas, increased TERC and TERT expression are associated with decreased ΟS in high-grade tumors [81]. Also, in cerebellar glioblastomas TERT promoter mutation and/or EGFR amplification, ATRX loss, and ALT pathway activation have been reported as independent prognostic factors with ATRX loss of expression, and ALT positivity to associated with a better outcome and TERT/EGFR alterations with a worse outcome of the patients [82].

Shelterin protein expression is also of prognostic and predictive significance for gliomas. Higher-grade gliomas are characterized by increased expression of ACD, a protein known for its involvement in shelterin complex assembly and interaction with telomerase. Upregulation of ACD is associated with poor prognosis and radiosensitivity in glioblastoma [83]. Furthermore, expression of POT1 (protection of telomeres protein 1), another shelterin component, and telomere length are also independent predictors of poor response to photon irradiation for GBM patients [84]. Exome sequencing of glioma patients from families with multiple affected members has identified rare inherited mutations in POT1 as high-penetrance glioma risk factors [85].

## Significance of Telomere Biology in Gliomas Treatment

Thousands of genes, among which telomere-regulating genes, have been found to affect sensitivity to temozolomide (TMZ), the standard chemotherapeutic treatment for glial tumors [86]. Telomerase, as one of the main contributors to telomere maintenance, was one of the first to be targeted for treatment purposes in GBM [87]. It is well known that telomerase is involved in TMZ resistance in GBM [88]. GRN163L (Imetelstat) is a common telomerase antagonist capable of crossing the BBB to shorten telomeres and induce cell cycle arrest and cell death, increasing survival [89–91]. It also shows synergistic effects with TMZ and radiation therapy [90]. Similarly, the synergistic effect of TMZ combined with the telomerase inhibitor BIBR1532 in vitro, based on a bioinformatical analysis, exhibits anti-proliferative properties in glioma cell lines on its own and especially in combination with TMZ [86]. Interestingly, several telomere or telomerase-targeting treatments have shown effect on tumor cell lines or animal models without affecting telomere length. After short-term treatment with the telomerase inhibitor MST-312, glioma cell lines undergo cell cycle arrest and apoptosis but subsequent adaptations after long-term treatment lead to increased aggressiveness [92]. MST-312 has also been shown to reduce pediatric ependymoma cell populations harvested from surgical specimens [93].

In addition, utilization of modern gene editing tools, such as CRISPR (Clustered Regularly Interspaced Short Palindromic Repeats) methodologies and antisense oligonucleotides pave the way for the application of gene therapy. These techniques, although they are still limited to preclinical studies, are a great example of the feasibility of precise correction of TERT mutations to inhibit CNS tumor growth [94] or hTERT knockdown via AON-Ex726-mediated targeting of the hTERT pre-mRNA splicing mechanism [95]. Promising approaches to TERT targeting for brain tumors including glioblastomas are very thoroughly reviewed recently by Patel et al. [47].

The inhibition of TRF1, a protein of the shelterin complex which is upregulated in GBM by multiple pathways [96], has been detected to impair tumor growth in GBM mouse models without disturbing normal brain function [96, 97]. G quadruplex stabilizers at telomeric ends—with telomestatin being the most representative—have also been studied as therapeutic targets in brain tumors. Specifically, glioma tumor cells are more sensitive to telomestatin than the normal brain parenchyma [98]. In addition, the acridine derivative BRACO-19—one of the most effective and specific ligand for telomeric G4—has been shown to disassemble telomere binding and shelterin proteins leading to telomere damage and reduced glioma cell growth, without affecting normal astrocytes [99]. Another interesting mechanism is that of the G quadruplex ligand CX5461 which reduces telomerase activity by interfering with its transcript splicing process. In this way, GBM cell lines undergo cell cycle arrest and a decrease in their proliferation [100]. Finally, G quadruplex stabilizers may have a synergistic effect with irradiation, as is the case of pentacyclic acridine (RHPS4) in glioblastoma cells [101], whereas TAC, a G-quadruplex ligand, reduces previously radioresistant GBM masses [102].

Arsenic has specific telomere-binding properties and thus arsenic-based compounds constitute another studied strategy. Arsenic trioxide generates reactive oxygen species (ROS) that activate DNA damage response at the telomeres and reduce telomerase activity, thus causing glioma cell senescence, apoptosis, and reduction of their migration and invasion [103]. Previously, Woo et al. had suggested that sodium meta-arsenite (KML001) affects telomere length, as they observed DNA-damage mediated apoptosis and reduction of xenograft tumor burden in combination with temozolomide and irradiation, without systemic toxicities in glioblastoma cells [104].

A common problem in cancer treatment is its recurrence after initial remission. A common mechanism of glioma recurrence is mediated by glioma stem cells (GSCs), a group of non-dividing chemoresistant cells which may however be reactivated after tumor removal. TRF1 [75] and TRF2 [105] inhibition can reduce stemness and increase survival in xenografted mice. Telomestatin selectively dissociates TRF2 from GSCs telomeres leading to telomeric DNA damage in GSCs but not in non-stem glioma cells (NSGCs) [106] and introduces telomeric and non-telomeric DNA damage in the context of failed repair mechanisms, thus reducing their self-renewal capacity and survival in vitro [98]. Similar results in terms of cell viability occur after the introduction of the nucleoside 6-thio-2′deoxyguanosine in stem cells derived from pediatric brain tumors including diffuse gliomas [107]. Recently, low-intensity pulsed ultrasound, a non-invasive method in human use, increased GSC sensitivity to temozolomide in vivo and in vitro by converting oxygen molecules to singlet oxygen that induces telomere shortening [108]. Importantly, recent data associate the ALT pathway with elevated glycolytic flux and demonstrate the ability of [6,6′-2H]-glucose to non-invasively assess tumor burden and response to therapy in astrocytomas [109].

A few research efforts targeting the telomere maintenance mechanism have been translated into early-phase clinical trials in gliomas, with limited success so far [87]. Specifically, NCT01836549 was A phase II clinical trial of imetelstat in various recurrent pediatric CNS tumors, which had to be prematurely terminated, following the death of two of 42 patients due to thrombocytopenia-related intracranial hemorrhage [110]. A telomerase 540–548 peptide vaccine in combination with the granulocyte–macrophage colony-stimulating factor sargamostim has reached phase I studies for adult and pediatric brain tumors and sarcoma but has not yet posted results (NCT00069940). Two additional ongoing trials utilizing telomere-related technologies are worth mentioning: NCT03491683 and NCT04280848. The former is investigating the biological agent INO-5401 comprised of three DNA plasmids each targeting a specific gene in glioblastoma patients, including hTERT. This agent is to be used in combination with another DNA plasmid expressing IL12 and an anti-PD1 monoclonal antibody. NCT04280848 on the other hand is a Phase 2 trial for GBM evaluating UCPVax, a cancer vaccine which relies on the introduction of two hTERT-derived peptides to activate CD4 + T-cells against cancer and have already shown to be safe and highly immunogenic in recent early-phase trial on non-small cell lung cancer [111].

## The Role of Glia Telomere Shortening in Traumatic Brain Injury

Traumatic brain injury (TBI) includes a vast category of neurological conditions. The main mechanism of TBI is external mechanical forces to the brain and is of major concern in adolescents of both sexes [112]. TBI is most manifested as mild traumatic brain injury (mTBI) and repeated mTBI (RmTBI), also known as concussion. mTBIs and RmTBIs have been associated with persisting cognitive deficits and increased incidence of neurodegenerative disease [113, 114]. The primary traumatic incident is followed by a secondary injury comprising a neurometabolic cascade which interferes with the initiation of an acute inflammatory response and oxidative stress as well as activation and infiltration of microglia [115, 116].

Relevant literature documents a positive correlation between telomere shortening and mTBI [117, 118]. Specifically, shorter telomeres have been associated with poorer performance in contrast to longer telomeres which have been associated with better performance in juvenile rats following a concussion/mild traumatic brain injury [117]. Furthermore, findings from an injury model of RmTBI in adolescent rats demonstrate a link between RmTBI and significantly shorter telomere length independent of sex [118]. More data highlight the presence of worse sensorimotor deficits and shorter telomeres, in brain specimens of middle-aged TBI rats compared to young ones [119]. However, it is worth mentioning that molecular mechanisms pertaining to telomere maintenance are active in the injured brain since microglia can maintain telomere length via telomerase during periods of high proliferation in vivo [120].

Additional research conducted by Hehar and Mychasiuk demonstrates a strong correlation between telomere length in brain tissues and peripheral skin cells which provides easily obtained samples [117]. It remains to be elucidated if there is a potential clinical use of telomere length as an injury prognosis biomarker. Although recent data are promising for the use of telomere length as a non-invasive biomarker for TBI, RmTBI, and concussion prognosis [117, 118], there are still obstacles to overcome in both pre-clinical models and humans before validated clinical results are acquired. Longitudinal studies with human populations should examine the baseline of telomere length since literature indicates telomere length is responsive to environmental manipulations and inter-individual factors such as prenatal stress [121] and dietary intake [122]. In accordance with this latest finding, a molecular analysis of Mychasiuk et al. demonstrated that diet-induced epigenetic pathways exist and are associated with differences in individual predisposition and resiliency to post-concussion syndrome [122].

Furthermore, vitamin E, an antioxidant agent, has been thought to improve neurological recovery after brain injury and inhibit functional deterioration in aging. More specifically, vitamin E has been shown to increase microglia cell proliferation in vitro providing potential benefits of vitamin E supplementation on microglial renewal capacity during aging or after brain injury [123]. However, these results require further investigation before the effectiveness of vitamin E supplementation in vivo is proven.

## The Role of Glial Telomere Length in Psychiatric Diseases

Two glia cell populations (oligodendrocytes and astrocytes) are characterized by differential resilience to biological stressors such as oxidative stress, known to play a role in accelerating telomere shortening [124]. Particularly, telomere lengths in white matter oligodendrocytes, but not astrocytes, from two areas of the brain, the frontal (Brodmann area 10) and temporal lobe (uncinate fasciculus), shown previously to demonstrate pathology in MDD patients, are significantly shorter in major depressive disorder (MDD) patients as compared to matched control donors suggesting a link between telomere shortening and white matter abnormalities described in MDD [124]. In addition, telomere length was found to be significantly reduced in oligodendrocytes from occipital cortical white matter and the immediate region of the locus coeruleus (LC) in the brainstem of MDD donors died by suicide as compared to control donors, in a study of Chandley et al. [125]. Likewise, although no difference of telomere length in the cerebellar gray matter derived from patients with MDD, bipolar disorder (BD), and schizophrenia, (SZ) compared to controls, was found; the telomere length has been detected as significantly shorter in MDD than in BD, SZ, and controls, in a study of Zhang et al. [126]. Moreover, Kronenberg et al. suggested a link between classical microglia activation with repression of telomere-associated genes in animal models of stroke, Alzheimer’s disease, and chronic stress. However, the effects of chronic stress in microglia activation were modest [127].

**Fig. 3:**
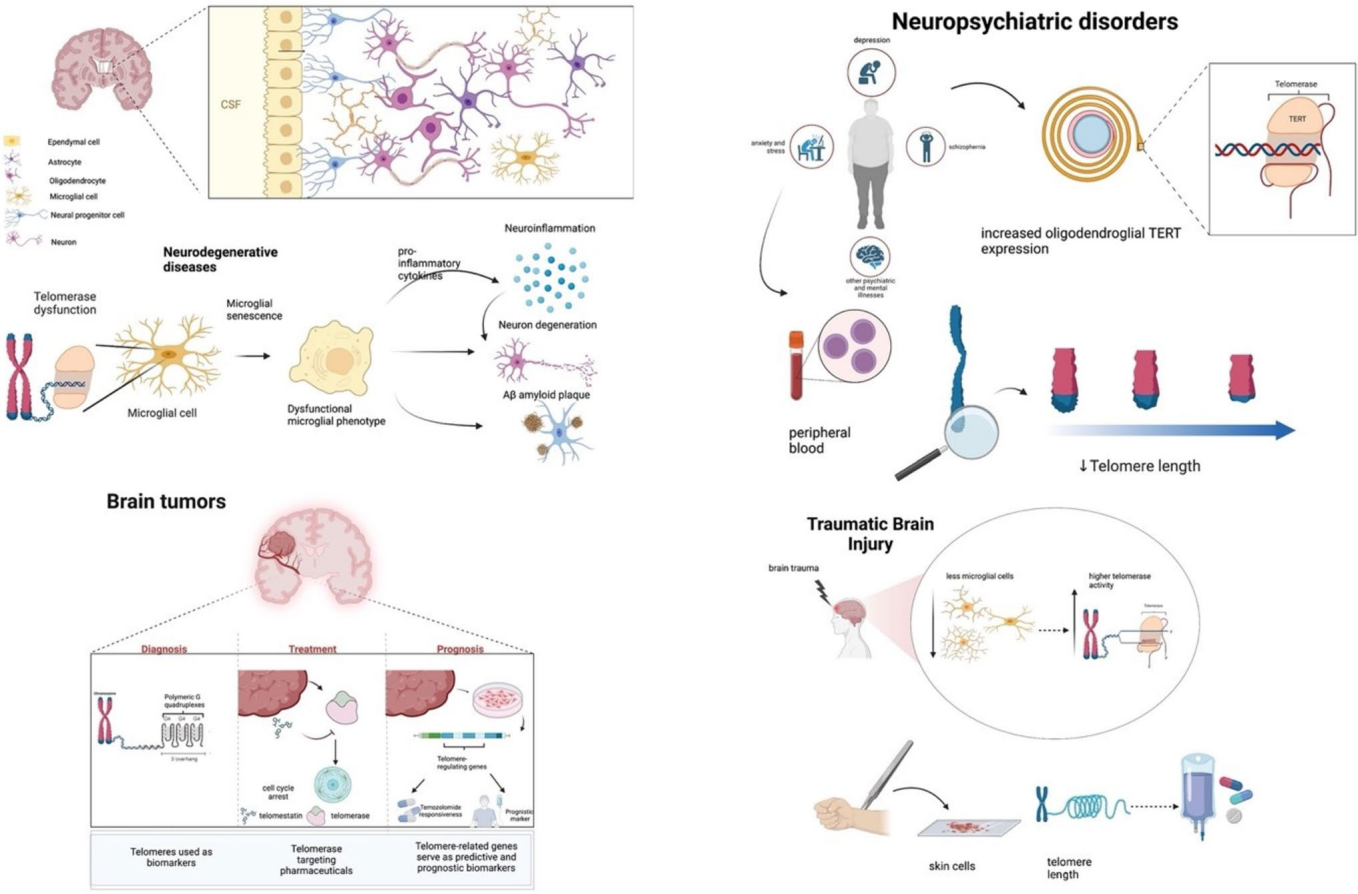
Neurodegenerative diseases: Top: schematic illustration of the topological relation between the glial cells and neurons in the CNS. Bottom: telomere shortening leads to microglial shortening and neuroinflammation; Brain tumors: Telomere-related biomarkers aiding in diagnosis, treatment, and prognosis of brain tumors; Psychiatric disorders: Glial telomere shortening is linked to various psychiatric disorders; Traumatic brain injury: Glial telomere length as a biomarker in traumatic brain injury

Previous studies have documented an abundance of shortened telomeres in peripheral blood samples (leukocytes and lymphocytes) of psychiatric patients as reviewed by Lindqvist et al. [128]. Nevertheless, further longitudinal studies in the human brain tissue, even though the limitations, are needed to enrich the knowledge concerning the relation between glial cells, telomere shortening, and possible clinical interventions in the field of psychiatric diseases. The role and implication of glial cell telomeres in neurodegenerative, and psychiatric diseases, gliomas, and traumatic brain injury are summarized in Fig. 3.

## Conclusion

Although the exact role of glial telomere shortening in brain pathogenesis is—in many cases—yet to be elucidated, ongoing research is bound to offer new perspectives promoting glial cells as key elements in brain physiology and pathophysiology. Furthermore, the already established complex interplay between microglia, other glial cells, as well as neurons in their respective niche reiterates the need to analyze the interactions between cells in their microenvironment to dissect disease pathogenesis. Additional research is needed to detect the extent of telomere shortening and senescence in both glial and neuronal tissue and thus analyze their role in CNS pathology. Mapping the connectome between different cell phenotypes comprising the neural tissue and investigating their molecular state in health and disease will provide further insights in our quest to illuminate the substrate of CNS disorders in the era of precision medicine.

## Data Availability

Not applicable.

## Abbreviations

ACD: Adrenocortical dysplasia protein homolog
AD: Alzheimer’s disease
α-KG: α-Ketoglutarate
ALT: Alternative lengthening of telomeres
AON: Antisense oligonucleotide
APB: ALT-associated promyelocytic leukemia bodies
ATRX: Alpha-thalassemia/mental retardation syndrome X-linked
BBB: Blood-brain barrier
CAT: Catalase
CNS: Central nervous system
CRISPR: Clustered regularly interspaced short palindromic repeats
DAXX: Death domain-associated protein
DDR: DNA damage response pathway
EGFR: Epidermal growth factor receptor
FUBP1: Far upstream element binding protein 1
GBM: Glioblastoma multiforme
GPX1: Intracellular glutathione peroxidase
GCN5: General control non-depressible 5
GSC: Glioma stem cells
IDH: Isocitrate dehydrogenase
KO: Knock-out
LC: Locus coeruleus
MDD: Major depressive disorder
MGMT: O6-methylguanine-DNA methyltransferase
mTBI: Mild traumatic brain injury
mTL: Mean telomere length
NFκB: Nuclear factor-κB
N-TASQ: Naptho-template assembled synthetic G-quartet
OS: Overall survival
PARK 2: Parkin gene 2
PARK 5: Parkin gene 5
PCAF: P300/CBP-associated factor
PD: Parkinson’s disease
POT1: Protection of telomeres 1
Rap 1: Ras-proximate-1 or Ras-related protein 1
RAP1: Ras-proximate-1
RmTBI: Repeated mTBI
ROS: Reactive oxygen species
SASP: Senescence-associated secretory phenotype
SOD1: Cytoplasmic superoxide dismutase
SOD2: Mitochondrial superoxide dismutase
SNCA: Synuclein gene
T/A: Relative telomere lengths
TBI: Traumatic brain injury
TERC: Telomerase RNA component
TERF2IP: Telomeric repeat binding factor 2 interacting protein
TERT: Telomerase reverse transcriptase
TIN2: TRF1-interacting nuclear protein 2
THOR: TERT hypermethylated oncological region
TMM: Telomere maintenance mechanism
TMZ: Temozolomide
TPP1: Tripeptidyl-peptidase 1
TRF1: Telomeric repeat-binding factor 1
T-SCE: Telomeric sister chromatid exchange
USP22: Ubiquitin-specific peptidase 22
XRCC1: X-ray repair cross-complementing 1
